# Effects of a PPAR delta agonist in a tauopathy mouse model

**DOI:** 10.1002/alz70859_104518

**Published:** 2025-12-26

**Authors:** Urmimala Raychaudhuri, Candice Tang, Harmanjeet Singh, Melika Seyedmohammadlou, Soo Jung Park, Matthew F Rose, Albert R La Spada

**Affiliations:** ^1^ University of California, Irvine, Irvine, CA USA

## Abstract

**Background:**

Elevated levels of ptau (phosphorylated tau) is directly correlated with neurodegeneration and neuropathogenesis in Alzheimer’s disease(AD). The presence of ApoE allele is a significant genetic risk factor for the progression of AD. PPAR‐δ, a nuclear receptor highly expressed in CNS neurons, regulates metabolic processes that helps boost mitochondrial function and improve quality protein control; processes that deteriorate as neurodegeneration progresses. This makes PPAR‐δ an appealing candidate for neurodegenerative diseases such as Alzheimer’s. Here we access the effect of PPAR‐delta agonist, T3D, on a mouse model of tauopathy P301S/E4 against a saline control group.

**Method:**

T3D was administered intraperitoneally at 50mg/kg/day, 3 days a week from ∼4 to ∼11 month of age. Neurological testing was performed every alternate month. Behavior studies such as Object Recognition Memory test (ORM), Object Location Memory (OLM), and Y maze studies were conducted at 7, 9 and 11 months.

**Result:**

The weight of T3D‐959 treated mice appear to deteriorate less compared to saline treated mice. Not much difference was seen in the overall neurological exam scores. In behavioral studies, T3D‐959 treated mice subjected to OLM performed the long‐term memory task significantly better compared to saline treated mice. Significant superior performance of the T3D‐959 treated mice were observed at 11 months of age (p=0.02, n=5 per group, two‐tailed t‐test; error bars = s.e.m.), indicative of better long‐term hippocampal memory function upon treatment with T3D‐959. There was no significant difference in the ORM and Y‐maze test.

**Conclusion:**

OLM testing evaluates dorsal hippocampus long term memory function and is transcription based. Y‐maze is non‐transcription based and studies short‐term memory function and spatial recognition. Several relevant brain regions, including the entorhinal cortex and ventromedial prefrontal cortex, are required for ORM. Significantly higher discrimination index (DI) scores were observed with OLM testing at 11 months of age in the T3D‐959 treatment group compared to saline, which demonstrates that T3D rescues hippocampal long term memory function better than saline. This is a potentially important finding that indicates that T3D‐959 can impact the hippocampus to preserve long term memory function.

